# Spinal Subdural Staphylococcus Aureus Abscess: case report and review of the literature

**DOI:** 10.1186/1749-7922-4-31

**Published:** 2009-08-06

**Authors:** Dimitris Velissaris, Diamanto Aretha, Fotini Fligou, Kriton S Filos

**Affiliations:** 1 Department of Anaesthesiology and Critical Care Medicine, Patras University Hospital, Patras, 26500. Greece

## Abstract

**Background:**

Only 65 cases (including our case) of spinal subdural abscesses have been reported to the literature, mostly to the lumbar spine. Staphylococcus aureus is the most common bacterial. The symptoms are not caracteristic and contrast – enhanced magnetic resonance imaging scan (MRI) is the imaging method of choice. The early diagnosis is crucial for the prognosis of the patient.

**Case presentation:**

We present a patient 75 years old who had a history of diabetes and suffered acute low back pain in the region of the lumbar spine for the last 4 days before his admission to the hospital. He also experienced lower leg weakness, fever and neck stiffness. After having a brain CT scan and a lumbar puncture the patient hospitalized with the diagnosis of meningitis. Five days after his admission the diagnosis of subdural abscess secured with contrast – enhanced MRI but meanwhile the condition of the patient impaired with respiratory failure and quadriplegia and he was admitted to the ICU. A laminectomy was performed eight days after his admission into the hospital but unfortunately the patient died.

**Conclusion:**

Early diagnosis and treatment are very important for the good outcome in patients with subdural abscess. Although morbidity and mortality are very high, surgical and antibiotic treatment should be established as soon as possible after the diagnosis has secured.

## Background

Spinal subdural abscess (SSA) is a very rare entity. Its exact incidence is unknown and to date only 64 cases have been reported in the literature [1]. Staphylococcus aureus (staph aureus) is the most common bacterial source [1-3] and thoraco – lumbar spine is the most affected region [1,2,4]. MRI is the diagnostic modality of choice. The first subdural empyema was reported in 1927 [5]. Bacterial abscesses involving spinal canal are associated with high morbidity and mortality, while early diagnosis and emergent treatment are vital to prevent the formation and progression of neurologic deficits and death. In this report, we present a patient with SSA in the thoracic and lumbar region.

## Case presentation

A 75-year-old man with a past medical history of diabetes mellitus was admitted to the Emergency Department of our University Hospital. He had a history of acute low back pain in the region of the lumbar spine in the last 4 days before his admission to the hospital. Two days before his admission he experienced lower leg weakness and fever (oral temperature 38.5°C). Clinical examination showed neck stiffness. After initial evaluation and brain CT scan – which revealed no damage – he had a lumbar puncture. The patient hospitalized with the diagnosis of meningitis (CSF: 765 white cells per cubic millimeter, elevated protein level: 70 mg per deciliter, decreased CSF glucose levels: 35% of serum glucose). Staph. aureus was cultured from cerebrospinal fluid (CSF) sample.

**Figure 1:**
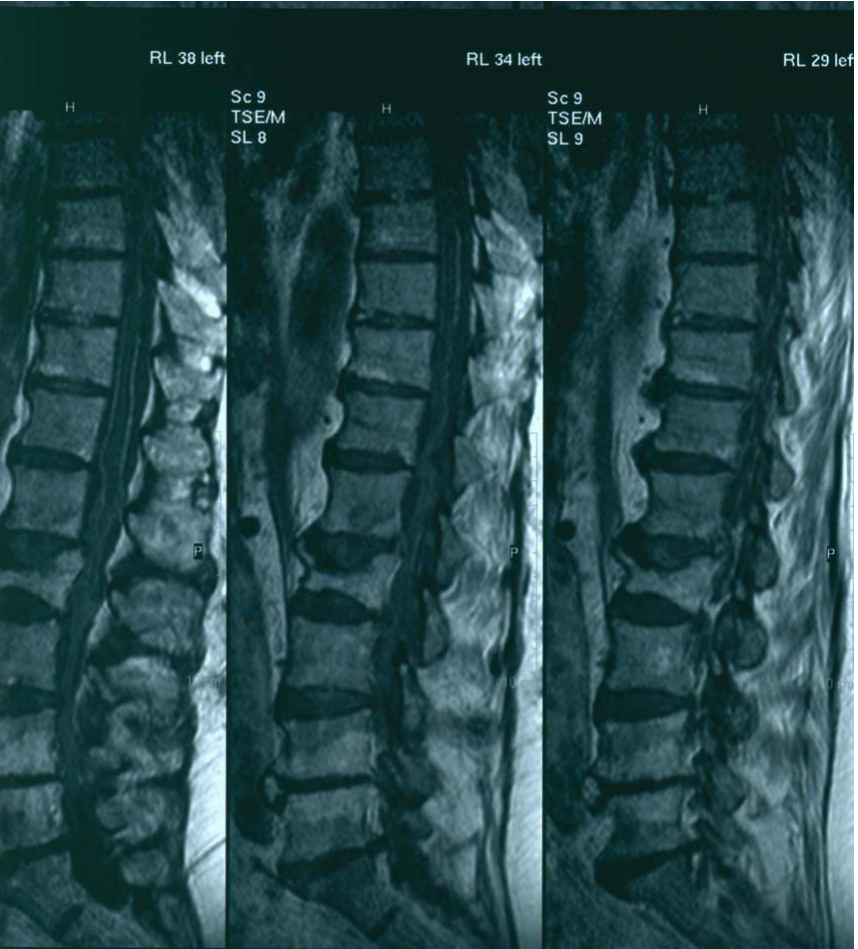
**Magnetic Resonance Imaging scan (MRI) mainly of the lumbar and partially of the thoracic spine**. Saggical scan.

**Figure 2:**
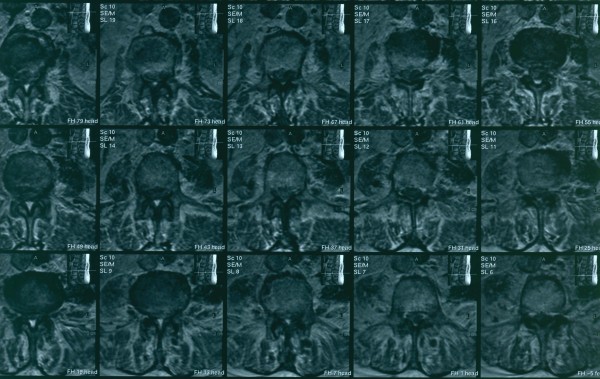
**Magnetic Resonance Imaging scan (MRI) of the lumbar spine**. Axial scan.

The neurologic condition of the patient impaired very quickly and at the end of the third day, after his admission, he developed paraplegia. Deep tendon reflexes were absent in the lower limbs and severely diminished in the upper limbs. After neurosurgical consultation an emergency magnetic resonance imaging scan (MRI) of the brain and the whole spinal spine was performed, five days after the admission of the patient to the hospital. It revealed a contrast-enhancing subdural mass collection posterior and left lateral to the spinal cord at the level L_2 _– L_4 _which was compressing the spinal cord. It also revealed arachnoiditis in the whole thoracic and lumbar vertebral body of the spinal cord. After intravenous contrast administration there was an intense enhancement on the boundaries of the collection and widespread meningeal enhancement (figures 1 and 2). Brain MRI with intravenous contrast revealed no intracranial abnormalities.

Meanwhile, at the end of the fifth day, the condition of the patient impaired with respiratory failure and quadriplegia and he was admitted to the ICU. The patient remained alert and cooperative. Laboratory data showed a leukocytosis of 20,000/mm^3 ^with a left shift, median elevated serum alkaline phosphatase (789 IU/l) and decreased albumin (2.8 g/dl). Also the C-reactive protein was elevated (17.5 mg/dl).

A L_2_–L_4 _laminectomy with midline incision of dura and arachnoid was performed eight days after the admission of the patient into the hospital. The purulent material of the abscess was observed posterior and left lateral to the spinal cord and unfortunately extended in the whole lumbar vertebral body of the spinal cord (according to the surgeon, there was possibly an empyema to the whole vertebral body of the spinal cord). An empyema was extended to lumbar nerve roots and to the psoas muscles. The purulent material was removed at the levels of laminectomy and the vertebral body copiously irrigated superiorly and inferiorly with saline solution. The wound was closed, and a usual drainage system was placed (inflow/outflow drain). Cultures from the purulent material and the blood were positive for staph. aureus.

Despite the removal of the purulent material and the appropriate antibiotic treatment (IV vancomycin, meropenem, fluconazole) the neurologic condition of the patient declined immediately after the operation and he developed severe impairment of consciousness. Except respiratory failure, which was always a problem, hemodynamic instability was also reported during his ICU stay. In ICU, all failure systems were supported. The patient was well hydrated, he was fed with enteral nutrition and he had an early tracheostomy in an attempt of weaning from mechanical ventilation. Inotropic and vasoactive agents were needed to stabilize mean arterial pressure >65 mmHg. The patient died 6 weeks after his ICU admission.

## Discussion and review of the literature

Spinal subdural abscess is very rare and its exact incidence is unknown, to our knowledge [1]. To date, including our patient, only 65 cases have been reported [1-4,6-19]. Articles, reviews and case reports published in English language journals and indexed by Pubmed (National Library of Medicine) were systematically searched. Additional articles and/or case reports were retrieved from the reference lists of the online found literature. A combination of the following terms was used in the search: spinal infection, spinal subdural abscess, Staphylococcus aureus and abscess. Cerebral abscesses, which are also extremely rare complications of infections (meningitis, pharegeal infection, sepsis, mastoiditis) or complications of rare syndromes/diseases, are not included in our review.

The review of these 65 case showed that staph. aureus was the most frequent causative agent (table 1) and the lumbar region the most frequent localization of the SSA (table 2). The most frequent age is between 60 and 70 years. It is a very uncommon localized central nervous system infection [1,20].

**Table 1:** Causative pathogen in the 65 cases of spinal subdural abscess

**Organism**	**Cases (number)**
*Staphylococcus aureus*	34
*Hemolytic streptococcus*	2

*Escherichia coli*	2

*Staphylococcus epidermidis*	1

*Pseudomonas aeruginosa*	1

*Streptococcus milleri/Fusobacterium sp./Streptococcus viridans*	1

*Diplococcus pneumoniae*	1

*Mycobacterium tuberculous*	2

*Peptococcus magnus*	1

*Streptococcus intermedius*	1

*E. Coli/Bacterioides vulgatus*	1

*S. aereus/S. viridans*	1

*S. viridans*	1

*Sterile*	3

*Unknown*	13

***Total***	**65**

**Table 2:** Spinal subdural abscess. Location in 65 patients

***Region of abscess***	***Cases (number)***
*Lumbar – L*	*19*
*Thoracal – T*	*11*
*Thoracolumbar – TL*	*9*
*Cervical – C*	*9*
*Cervicothoracal – CT*	*4*
*Cervicothoracolumbosacral – CTLS*	*2*
*Thoracolumbosacral – TLS*	*3*
*Lumbosacral – LS*	*3*
*Cerebral+whole spine – C+Sp*	*3*
*Cervicothoracolumbar – CTL*	*1*
*Sacral-caudal – SC*	*1*
*Total*	*65*

Most patients with spinal subdural abscess have one or more predisposing conditions [1,3,21], such as an underlying disease which diminishes resistant of the patient to infection (diabetes mellitus, alcoholism, tumors or infection with human immunodeficiency virus), anatomical abnormalities of the spinal cord or vertebral column or intervention [17,22] (degenerative joint disease, trauma, surgery, drug injection, placement of catheters or stimulators). The development of SSA could be secondary to hematogenous spread of infection from an other region [23], infected CSF and direct spread into the subdural space [24], hematogenous inoculation during the course of meningitis [24], secondary inoculation due to lumbar puncture, direct contact with intraspinal space (osteomyelitis) and secondary infection after spinal surgery [24-26]. There are only two cases of SSA in the literature that are unrelated to such conditions and without well documented etiology [8].

Back pain at the level of the affected spine, fever and neurologic deficits such as para/tetraparesis, bladder dysfunction, disturbances of consciousness and inflammatory signs are some typical symptoms of SSA [3,4,20]. An established staging system for abscesses outlines the progression of symptoms and physical findings: stage 1, fever with or without spinal or nerve root pain; stage 2, mild neurological deficits are added to the clinical picture; stage 3, paralysis and complete sensory loss occur below the level of the lesion [27]. Our patient demonstrated low back pain, paraparesia and in the course of the time he developed paraplegia and quadriplegia. He developed stage 3 symptoms.

The most common causative agent is Staph. aureus and some predisposing factors are alcoholism, diabetes mellitus, immunosuppressive drugs, malignant tumor, chronic renal failure, intravenous drug abuse, rheumatic heart valve disease and tuberculosis. In this case report SSA developed in our patient, possibly, as a complication of meningitis in a background of a chronic disease such as diabetes mellitus. In our patient the causative agent was Staph. aureus. The patient revealed involvement of the central neural system which may result a poor outcome.

MRI, myeloCT, and computerized tomography (CT) are the most common diagnostic modalities. Contrast – enhanced MRI is the imaging method of choice because it is less invansive and due to its superiority in sensitivity in detecting the exact location and extension of the abscess which is essential for planning surgery [1,3,5]. MRI is also the modality of choice for diagnosing compressive myelopathy [28].

Leukocyte count, erythrocyte sendimentation rate (ESR) and C- reactive protein, although usually are found elevated, are not sensitive indicators of spinal infections [17,29,30]. Our patient had a leukocytosis of 20,000/mm^3 ^with a left shift and elevated C – reactive protein (17.5 mg/dl).

Surgical drainage together with systemic antibiotics is the treatment of choice [1,2]. Without intervention, stage 3 symptoms would develop and surgery performed after this stage may not reverse the neurological deficits. Unfortunately, our patient developed stage 3 symptoms before surgical intervention. Laminectomy, sometimes in more than one level depending of the extension of the abscess, could be necessary. When laminectomy in more than three levels is necessary this could result in spinal instability [1,31] Because the rate of progression of neurologic impairment is difficult to predict and some patients became paralyzed within hours after the onset of neurologic deficit, laminectomy, evacuation of the pus-like material and debridement of infected tissues should be done as soon as possible [1,3]. Outflow or inflow/outflow drainage systems could be used and be very useful. In cases of wider spread a single laminectomy in several different levels could be performed. Postoperatively a second spinal MRI should have been conducted, however the patient was hemodynamically unstable, with respiratory deficiency and it was not safe for him to be transferred to the MRI room (which, in our hospital, is in a long distance from the ICU).

In our patient MRI and laminectomy performed 5 and 8 days respectively after the admission of the patient to the hospital, which is not 'as soon as possible'. The patient had a wider spread of the purulent material into the lumbar and perhaps the whole spine so perhaps a laminectomy at several different levels to evacuate the pus like material should have been done. Furthermore, during the lumbar puncture there is a risk (although rare) to spread the infected pus-like material if the needle traverses the abscess which could have happened to our patient. Unfortunately our patient had a poor prognosis and died 6 weeks after his admission in the ICU.

## Conclusion

Spinal subdural abscess is a very rare but well described entity and associated with high morbidity and mortality. It is a neurosurgical emergency and as soon as diagnosis is established surgical treatment in collaboration to antibiotic therapy should be performed. Progressive neurological deficits, severe pain and fever suggest the diagnosis. The timing of the contrast-enhanced MRI, which is the modality of choice, is very important when the physicians notice the above symptoms. Staph aureus should be considered the most possible pathogen.

## Competing interests

The authors declare that they have no competing interests.

## Authors' contributions

DV participated in the data collection in the analysis of the data, reviewed and revised the manuscript. DA participated in the data collection and prepared the manuscript. FF participated in the data collection and in the analysis of the data. KSF reviewed and revised the manuscript and has given final approval of the version to be published. All authors read and approved the final manuscript.

## Consent

Written informed consent was obtained from the patient relative for publication of this case report and MRI images. A copy of the written consent is available from the editor-in-chief of the journal.
